# Orthodontic Replacement of Lost Permanent Molar with Neighbor Molar: A Six-Year Follow-Up

**DOI:** 10.1155/2017/4206435

**Published:** 2017-11-29

**Authors:** Taisa Boamorte Raveli, Ricardo Lima Shintcovsk, Luegya Amorim Henriques Knop, Luana Paz Sampaio, Dirceu Barnabé Raveli

**Affiliations:** Department of Orthodontics, Faculty of Dentistry, São Paulo State University, Araraquara, SP, Brazil

## Abstract

Extraction is very frequent indication in orthodontic planning, especially when there are crowding, biprotrusion, and aesthetically unpleasant profiles. Next to extraction comes space closure, which represents a challenge for orthodontists because of extended treatment time, discomfort created for the patient, tissue tolerance, and stability concerns. When it comes to what mechanics to choose for space closure, loops present two major advantages in relation to sliding mechanics: absence of abrasion and possibility to reach pure dental translation. A case is presented where an adult female patient with early loss of the first lower permanent molars, minor lower crowding, and tooth biprotrusion was treated with upper first bicuspids extraction along with upper and lower space closure done with T-loops to promote best space closure control in order to correct the malocclusion and enhance facial aesthetics.

## 1. Introduction

First bicuspids extraction is very frequent indication in orthodontic planning, especially when there are crowding, biprotrusion, and aesthetically unpleasant profiles. These teeth are generally selected to extraction due to their position in the center of upper and lower arches and normally closer to the crowding area [1]. Extraction of permanent molars is also indicated for the correction of dental malocclusion, and depending on the case, it can reduce the treatment time and turn orthodontic mechanics more simple [1].

It is frequent in orthodontic practice to deal with adult patients who present early loss of permanent molars and require some type of orthodontic treatment [2]. The maintenance of this edentulous space results in alveolar bone atrophy interfering in space closure and further possibility of dental implants [3]. In these cases of early loss of first lower permanent molar, the orthodontic repositioning of neighbor teeth in the edentulous area has been proven to be an excellent treatment option [4, 5].

Space closure represents a challenge for orthodontists because of the extended treatment time, the discomfort created for the patient, tissue tolerance, and stability. Moving teeth without any inclination is the objective, which makes vertical control a major concern [6].

The wider surface of lower permanent molar roots hinders the closure of spaces and many times produces unpleasant tooth movement such as the lingual tipping of incisors. Therefore, the segment of the arch that serves as anchorage to the space closure must be in control in this type of treatment [7]. The movement of the lower permanent molars is even more complex when compared to upper permanent molar movement because mandible presents thick cortical bone and small trabecular bone. In addition, the roots of lower molars are wider in the buccal-lingual direction [8].

When it comes to what mechanics to choose for space closure, loops present two major advantages in relation to sliding mechanics: absence of abrasion and possibility to reach pure dental translation, that is, body movement without tipping, if a moment force ratio (M : F) of roughly 10 : 1 is obtained [9]. Kuhlberg and Burstone [10] demonstrated that the production of symmetric T-loop springs using titanium-molybdenum alloy (TMA) of 0.017 × 0.025 inches thick involves a M : F ratio of roughly 12 : 1 with an activation of 2.5 mm.

The aim of this paper is to report a clinical case of a patient with early loss of the first lower permanent molars that also presented tooth biprotrusion with an unusual orthodontic option employed for the correction of the malocclusion.

## 2. Case Report

**Figure 1:**
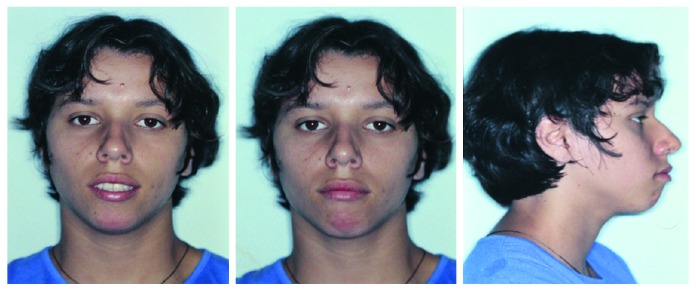
Pretreatment facial photographs (19 years, 0 month).

A nineteen-year-old female patient had been referred to the orthodontist by a general practitioner, and her major complaint was unsatisfactory facial and dental aesthetics in addition to remaining dental spaces due to early loss of the permanent first lower molars. Clinical records indicated no step back for orthodontic treatment. The extraoral examination pointed out to slight facial asymmetry, convex profile, and absence of passive lip sealing (Figure 1). No signs and symptoms of TMJ dysfunction were indicated.

**Figure 2:**
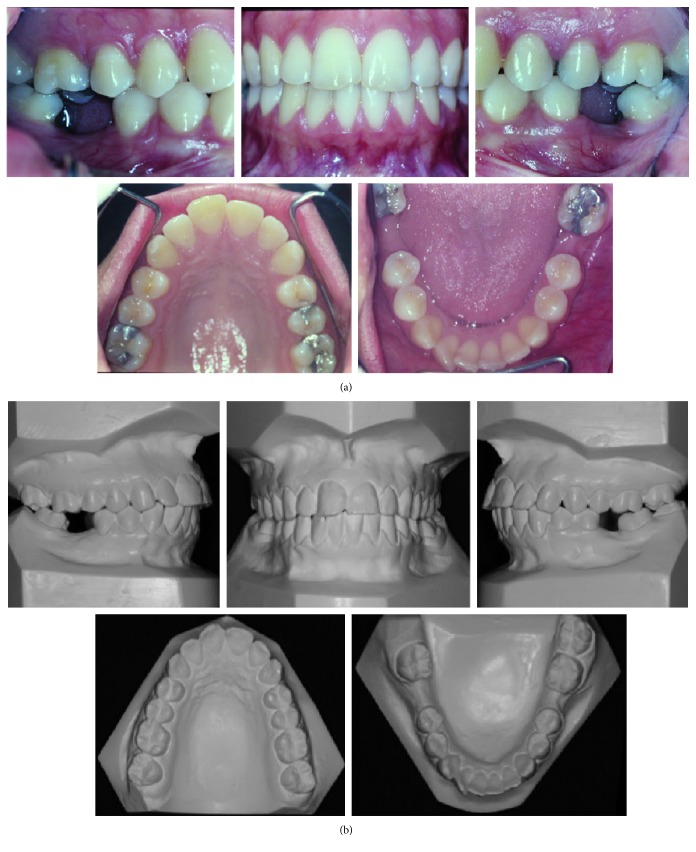
(a) Pretreatment intraoral photographs (19 years, 0 month). (b) Dental casts before treatment.

The intraoral clinical examination and casts evaluation revealed that the patient was in permanent dentition phase, with the absence of teeth 36 and 46, Class I canine relationship, and biprotrusion (Figure 2). The early loss of the first lower molars had occurred due to extensive cavities. The edentulous spaces presented 6 mm on the left side and 9 mm on the right side. The bucco-lingual width of the alveolar crest was 5 mm on the left side and 4 mm on the right side. The curve of Spee was moderate (Figure 2).

**Figure 3:**
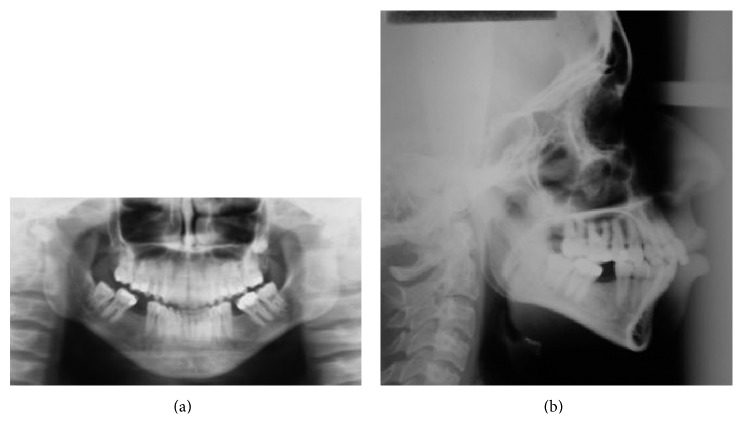
Pretreatment panoramic (a) and lateral (b) radiographs at 19 years, 0 month.

The panoramic radiograph examination showed absence of the first lower molars, tipping of the second molars towards the edentulous space, and presence of the left third lower molar and right third lower molar in the eruption process, with 2/3 of the root formed. No significant indication of bone loss in the edentulous region was indicated (Figure 3(a)).

The lateral cephalometric analysis indicated maxillary protrusion and minimum mandibular retrusion (Figure 3(b)). In addition, the dolichocephalic skeletal pattern was observed, proclined upper and lower incisors, which leads to biprotrusion diagnosis and protrusion of lower lip turning soft tissue profile not suitable (Table 1).

The objectives of this treatment were to improve facial aesthetics, correct dental biprotrusion, and close edentulous spaces.

The patient's complaint regarding her facial aesthetics could have been solved through the extraction of the upper and lower first bicuspids as well as anterior retraction if conventional orthodontics were to be applied. The space of the lower molars could have been maintained with further prosthetic rehabilitation using implants and/or fixed prosthesis. After discussing the treatment options with the patient, it was decided to close the edentulous lower spaces instead of extraction of first lower bicuspids and extract the upper first bicuspids in order to maintain canine Class I and provide anterior retraction on upper and lower arches.

A Roth prescription bracket, slot 0.022″ (Abzil, 3M), was installed, and bands were placed on the upper first molars and lower second molars and also on third lower molars. The initial alignment and leveling were carried out using 0.012″ NiTi, 0.014″ NiTi, and 0.016″ NiTi and 0.018″ and 0.020″ stainless steel wires until reaching 0.019 × 0.025″ ss. At this stage, the upper arch was segmented in three parts: (1) between canine-canine, (2) second bicuspid to first right molar, and (3) second bicuspid to first left molar. The lower arch was also segmented in three parts: (1) between second bicuspids; (2) second molar to third right molar, and (3) second molar to third left molar. This segmentation was done in order to prepare for installation of T-loops, and because a segmented arch technique was planned, this needed to be done.

**Figure 4:**
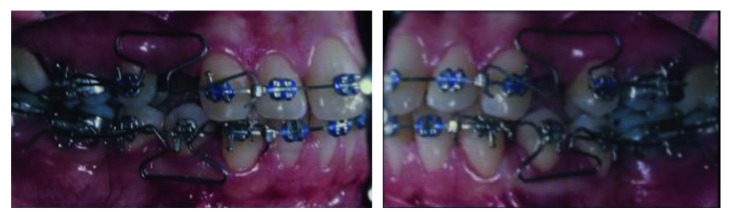
T-loop positioned for edentulous space closure. In maxilla, the T-loop was symmetrically positioned, and in mandible, it was positioned displaced for the anterior region.

T-loops were then constructed with 0.017 × 0.025 TMA wire and positioned on the upper and lower arches for space closure. The springs were positioned between the cross tube (lateral incisor and canine) and the accessory tube of the bands. The upper arch received a T-loop symmetrically activated, type B, to provide anterior retraction and anchorage loss at the same time. The lower arch, on the other hand, had a T-loop displaced to the anterior, type A, enabling anterior retraction (Figure 4) without or with minimum anchorage loss of second molars. The preactivations of the T spring were conducted according to Kuhlberg and Burstone [10].

After the full closure of spaces, details of the occlusion were taken into consideration with continuous 0.019 × 0.025″ stainless steel arches. After 32 months of active treatment, the fixed appliance was removed.

**Figure 5:**
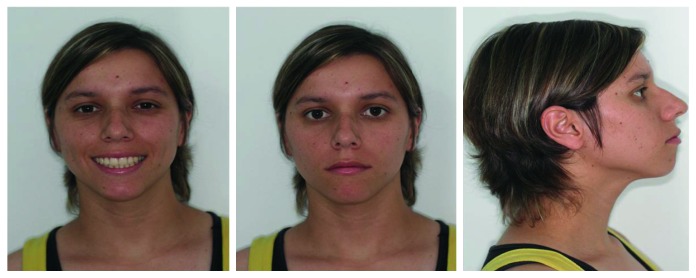
Posttreatment facial photographs (22 years, 1 month).

**Figure 6:**
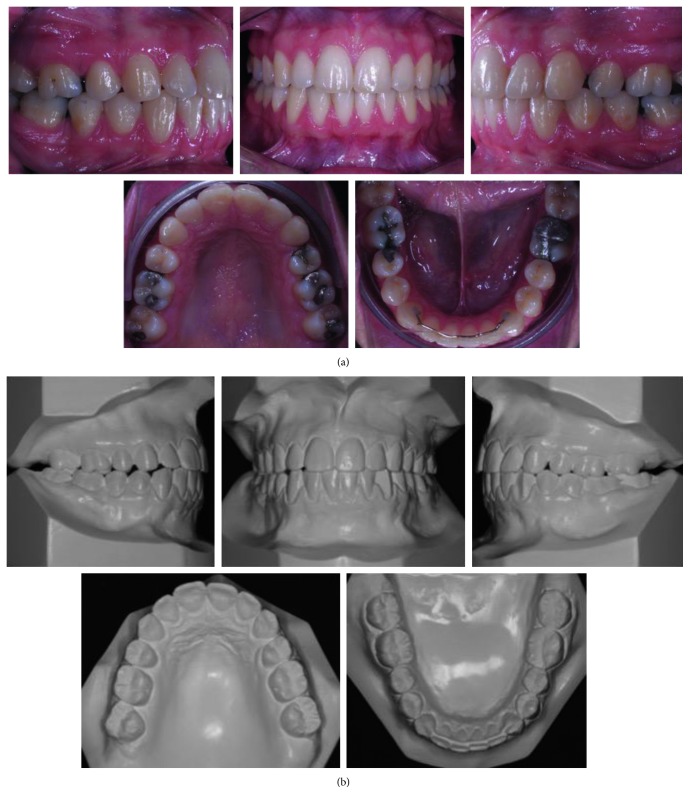
(a) Posttreatment intraoral photographs (22 years, 1 month). (b) Posttreatment dental casts.

**Figure 7:**
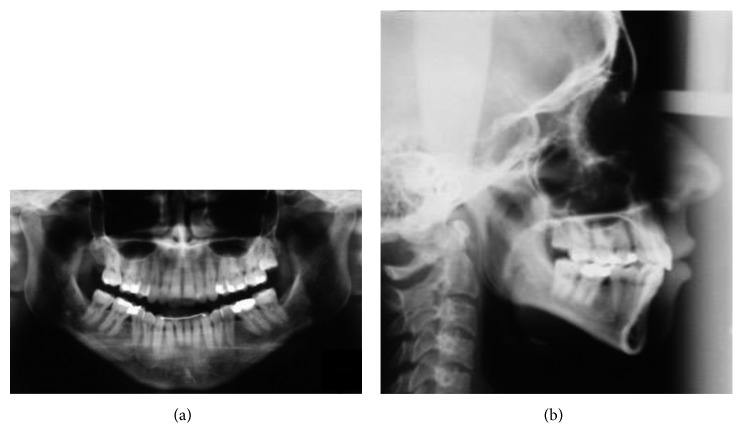
Posttreatment panoramic (a) and lateral (b) radiographs at 22 years, 1 month.

By the final stage of the orthodontic treatment, we observed more pleasant facial aesthetics with improvement of the lip protrusion and passive lip sealing (Figure 5). The intraoral examination pointed out to a satisfactory occlusion with coincidental mid-lines, Class I canine relationship, Class II molar relationship, and correction of upper and lower incisor excessive inclination occurred. The spaces of teeth 36 and 46 were fully closed. The major alterations were the retraction of the anterior teeth and space closure (Figures 6(a) and 6(b)). The panoramic radiograph pointed out average control of posterior lower teeth axial positioning and the presence of tooth 28 (Figure 7(a)). Lateral radiograph showed better inclination of upper and lower incisors, along with better lip sealing and improvement of soft tissue profile (Figure 7(b)).

Information shown in Table 1 shows the gradual increase of nasolabial angle through debonding and follow-up; decrease of IMPA, demonstrating the retraction of inferior incisors; and an improvement of interincisal angle and upper and lower aesthetic line.

**Figure 8:**
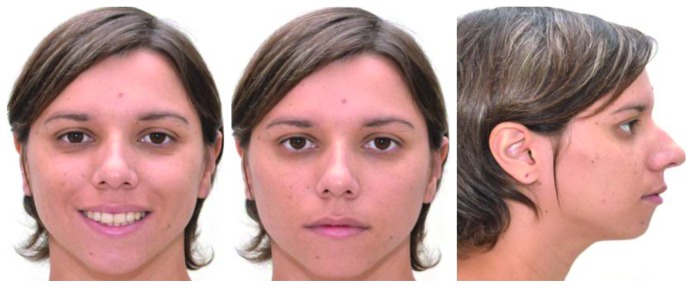
Six-year follow-up facial photographs (28 years, 8 months).

**Figure 9:**
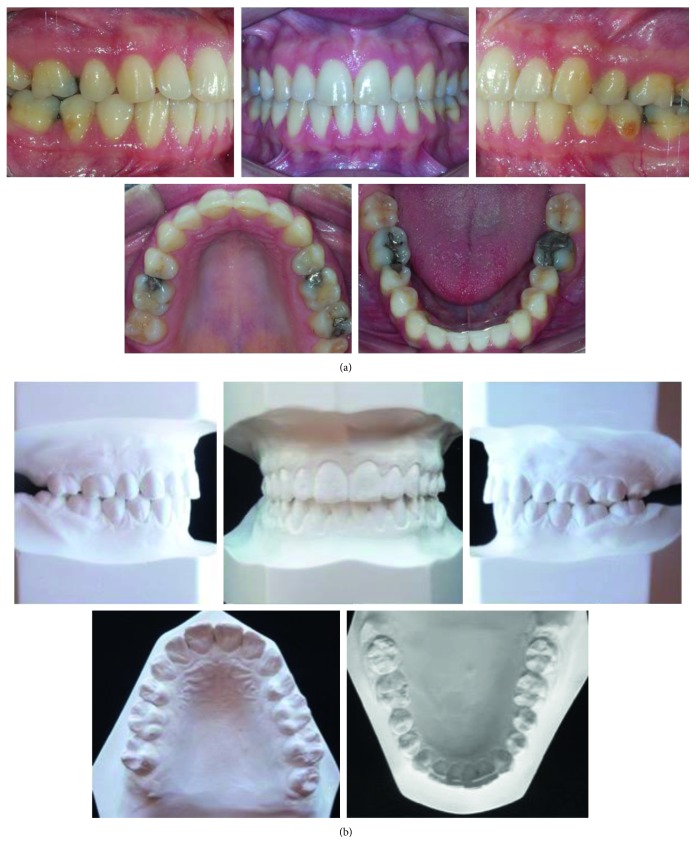
Six-year follow-up intraoral photographs (28 years, 8 months). (b) Six-year follow-up dental casts.

**Figure 10:**
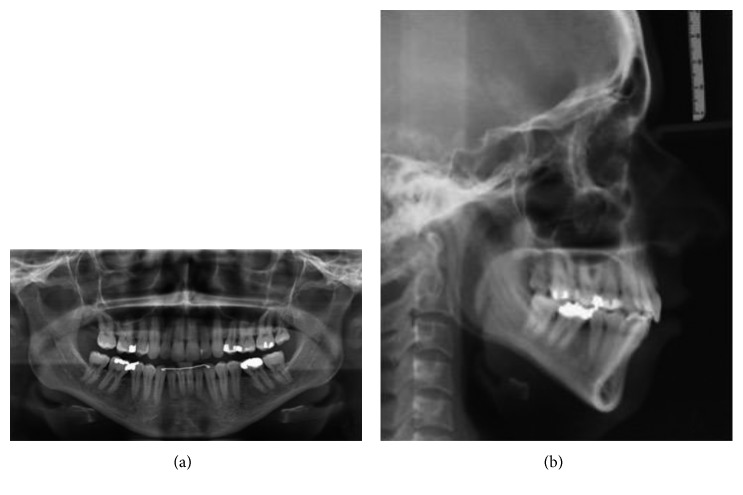
Six-year follow-up panoramic (a) and lateral (b) radiographs at 28 years, 8 months.

**Figure 11:**
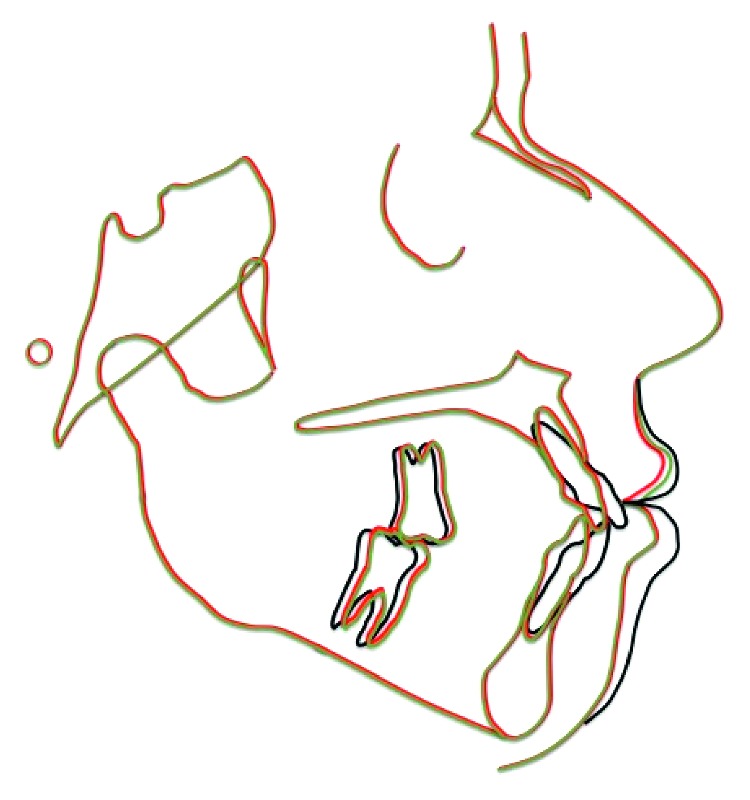
Superimpositions of the lateral cephalograms between the beginning, debonding, and 6-year follow-up stages: black line (19 years, 0 month); green line (22 years, 1 month); red line (28 years, 8 months).

The facial characteristics obtained after the treatment were maintained throughout a six-year follow-up (Figure 8). Extraction spaces remained closed, and little alteration occurred in the positioning of the incisors (Figures 9(a) and 9(b)); panoramic and lateral radiographs demonstrated that the characteristics were maintained (Figures 10(a) and 10(b)). The superimposition of cephalometric tracings can show dental and profile modifications during orthodontic treatment through a 6-year follow-up (Figure 11).

## 3. Discussion

The literature approaches different treatment options for the loss of first lower permanent molars [2, 7, 11]. The autotransplant is a good option when preservation is taken into account with teeth and their inborn periodontal structure, requiring no artificial material; however, this procedure may result in surgical trauma, root resorption, ankylosis, and infection, with variable success rates [11]. A fixed prosthetic is another option for these patients, but with a few limitations such as cost, partial abrasion of basic tooth structures, secondary decays, and mechanical errors [7]. In several cases, the decision is for implants.

Another method to treat the loss of first molars is the orthodontic respositioning with neighbor teeth [2, 4, 7, 12]; this possibility exempts the patient from surgical trauma and costs with the installation of implants or prosthesis. Furthermore, if the patient requires the correction of other orthodontic problems, the treatment will have minimum additional time [4]. According to Hom and Turley [12], the ideal dimensions for the closure of the lower molars spaces are 6 mm or less for the mesiodistal space and 7 mm for the bucco-lingual width.

In this clinical case, the patient sought orthodontic treatment for the correction of a dentoalveolar protrusion; since she had an early loss of the first lower molars, it was proposed extraction of upper first bicuspids and lower space closure with retraction of anterior teeth, utilizing the extraction already done in inferior arch instead of taking out first bicuspids.

The accurate control of the orthodontic movement during the closure of the extraction spaces is very important in orthodontic mechanics including control of anchorage units and vertical forces as well as axial tipping and rotations [9, 10].

Tip-back bends from Tweed mechanics, Begg or Tip-Edge mechanics, and intermaxillary or extraoral elastics may cause modification in the moment force ratio between anterior and posterior teeth [10]. However, the use of extraoral and intermaxillary elastics may not control the differential horizontal movement since the patient's collaboration is required [9].

When employing a T-loop from the segmented arch technique, it is possible to produce different moments that will result in the desired force system according to the clinical scenario. If the T-loop is centrally positioned, equal and contrary moments will be produced with negligible vertical forces. While a decentralized T-loop generates a higher root translation/movement of the segment close to it, and the distant segment suffers tipping into the direction of the extraction area [10].

Based on this principle described by Kuhlberg and Burstone [10], the T-loops used in this clinical case were adapted to generate differential moments. In the upper arch, it was centrally positioned between the tubes since the retraction of the anterior teeth was required simultaneously to minimum anchorage loss. While the lower arch, on the other hand, had greater space, therefore, the T-loop was displaced to the anterior, and this way the posterior segment would suffer tipping and anterior translation when retracting. This way a canine Class I and molar Class II could be achieved.

Another method described to benefit space closure is the use of temporary anchorage devices including mini-implants and mini-plates. Nagaraj et al. [7] described a space closure case where the force for space closure was generated by Nitinol closed spring coil anchored in mini-screws positioned between bicuspids using 0.018 × 0.025″ stainless steel arches. Using chain elastic adapted between the lingual surface of molars and an accessory button placed on canines prevented rotation tendency of molars. The authors commented on the occurrence of a certain tipping of the lower incisors due to use of chain elastic. The result was that the second molars presented minimum root resorption with no indication of fenestration or bone dehiscence. Hom and Turley [12] also observed minimum root resorption of the lower molars.

Saga et al. [2] also demonstrated space closure of first lower molars through the protraction of the second lower molars into the area of atrophic bone crest. A modified helical loop was used in 0.018 × 0.025″ continuous arch in order to retract the incisors and simultaneously protract the second lower molars. In order to prevent lingual tipping of lower incisors, Class II intermaxillary elastics and vestibular torque in the anterior lower region were employed.

Similarly, in this clinical case, no areas of bone dehiscence/fenestrations or root resorption were observed at the final stage of the orthodontic treatment despite the occurrence of a certain level of vertical bone loss before tooth movement due to an early loss of the first lower molars. These clinical findings are in accordance with other authors [2, 7, 12].

In contrast, Stepovich [5] reported that, in adult patients compared with younger patients, the closure of spaces with the protraction of the first lower molars results in lower bone apposition on the narrow bone crest, poor maintenance of closed space, and in some cases root resorption.

A relevant point to observe from this case presented is final position of lower roots. Since second molars suffered some tipping when space was closed, their final position was not vertical. Instead, there was a little angulation toward the space closed. Root parallelism is considered an objective in general orthodontic treatment, for long-term stability purposes. However, there is a six-year follow-up record of the case showing that, even with nonparallel roots at the end of the treatment, teeth positions were stable for a six-year period of time.

## 4. Conclusion

It is possible to treat edentulous space in adult patients without implants or prosthetics, especially with a segmented arch technique using principles of differential moments of the T-loop. Correction of biprotrusion was achieved with extraction of upper bicuspids in association with retraction; no areas of bone fenestrations and dehiscence or root resorption were observed; therefore, this possibility provides an alternative treatment that is safe for patients presenting early loss of the first lower permanent molars.

## Figures and Tables

**Table 1:** Cephalometric measurements at initial, debonding, and 6-year follow-up stages.

Measurement	Mean	Initial (19 y, 0 m)	Debonding (22 y, 1 m)	6-year follow-up (28 y, 8 m)
SNA (°)	82	86.3	86.6	86.8
SNB (°)	80	79.6	80.7	81.1
ANB (°)	2	6.7	5.9	5.7
SN to mandibular plane (°)	32	41.5	41.5	42.5
U1 to SN (°)	103	115	96.5	98.5
IMPA (°)	87	107	89.8	89.5
Interincisal angle (°)	130	105	135	135
Upper lip to aesthetic line (mm)	1.0	3	1	1
Lower lip to aesthetic line (mm)	0.3	7	3	3.5
Nasolabial angle (°)	110	81.94	99.6	102.7
